# Intratumoral Treatment in Lung Cancer: Is It Time to Move Towards Clinical Practice?

**DOI:** 10.3390/cancers16233892

**Published:** 2024-11-21

**Authors:** Gabriele Giuseppe Pagliari, Francesca Colonese, Stefania Canova, Maria Ida Abbate, Luca Sala, Francesco Petrella, Thoma Dario Clementi, Diego Luigi Cortinovis

**Affiliations:** 1 Medical Oncology Unit, Fondazione IRCCS San Gerardo dei Tintori, 20900 Monza, Italy; francesca.colonese@irccs-sangerardo.it (F.C.);; 2 Medicine and Surgery Department, Milano Bicocca University, 20126 Milan, Italy; 3 Department of Thoracic Surgery, Fondazione IRCCS San Gerardo dei Tintori, 20900 Monza, Italy; francesco.petrella@irccs-sangerardo.it

**Keywords:** intratumoral therapies, locoregional treatments, lung cancer

## Abstract

According to a modern view, cancer no longer follows a purely mechanistic model. Rather, a tumor is conceived as a more complex structure, composed of cancer cells, the activities of which may interact and reshape the so-called tumor microenvironment (TME), leading to preservation of specific tumoral niches and promoting the survival of tumoral stem cells. **Background/Objective**: Therapeutic strategies must deal with this unique cancer architecture in the near future by widening their range of activities outside the cancer cells and rewiring a TME to ensure it is hostile to cancer growth. Therefore, an intratumoral therapeutic strategy may open the door to a new type of anticancer activity, one that directly injures the tumoral structure while also eliciting an influence on the TME through local and systemic immunomodulation. This review would like to assess the current situation of intratumoral strategies and their clinical implications. **Methods** We analyzed data from phase I, II, and III trials, comprehensive reviews and relevant clinical and preclinical research, from robust databases, like PUBMED, EMBASE, Cochrane Library, and clinicaltrials.gov. **Results:** Intratumoral strategies can be quite variable. It is possible the injection and inhalation of traditional antiblastic agents or immunomodulant agents, or intrapleural administration. Ablation strategy is available, both thermal and photodynamic method. Moreover, TTfields and NPs are analyzed and also brachytherapy is mentioned. Intratumoral therapy can find space in “adjuvant”/perioperative or metastatic settings. Finally, intratumoral strategies allow to synergize their activities with systemic therapies, guaranteeing better local and systemic disease control. **Conclusions:** Intratumoral strategies are overall promising. Antiblastic/immunomodulant injection and NPs use are especially interesting and intriguing. But, there is generally a lack of phase II and III trials, in particular NPs use need additional experimentation and clinical studies.

## Simple Summary

The therapeutic strategies of NSCLC focus on systemic strategies, mainly chemotherapy and immunotherapy. Unfortunately, cancer has some aces up it’s sleeve, in particular the tumoral microenvironment. An intratumoral strategy can be useful to overcome cancer’s shelters. Some intratumoral strategies have been analyzed and proved during the last decades, including a treatment based on “classical” antiblastic agents, immunotherapy, and immunomodulating agents, which can be administered by direct injection, inhalation, and intrapleuraly. Other treatments take advantage of ablation techniques, including photodynamic and thermal. Moreover, it is possible to evaluate the use of electric fields, a promising technique, and brachytherapy, a consolidated one. Finally, nanoparticles are interesting and useful both for their direct activity and for their carrier role. Each intratumoral strategy should be chosen based on setting, metastasis status, cancer type, and patient features, etc. Furthermore, the evaluation of a combined approach is fundamental, because intratumoral and systemic strategies together can overcome the other’s limitations and mutually enhance one another in order to become more efficient tools in the anticancer war.

## 1. Introduction

Lung cancer is a chronic worldwide plague, with as many as 2.1 million new diagnoses a year, and is the leading cause of cancer-related death, with up to 1.8 million deaths per year. Rates differ across the world as a function of variations in local/national risk factors, of which the most common cause is tobacco [1].

Non-Small Cell Lung Cancer (NSCLC) is usually diagnosed in the late stages, at which point systemic drugs are administered. Nowadays, however, there is a significant range of therapies available, such as chemotherapy, immunotherapy, targeted therapies, or a combination of different protocols. While chemotherapy has not proven to be a “silver bullet”, it remains a key treatment for many patients, despite its kill or cure cost in terms of its toxicity. Moreover, the penetrability of chemotherapy in tumors remains unclear. Indeed, some factors may reduce efficacy, such as abnormal tumor vasculature, elevated interstitial fluid pressure within the tumor, hypoxic intratumor and tumoral microenvironment, the presence of a dense extracellular matrix surrounding the tumor [2,3,4], and intratumoral necrosis.

The recent introduction of antibody drug conjugates (ADCs) in clinical practice has been aimed at facilitating the release of cytotoxic drugs directly within the tumoral cells, thus improving reach while reducing systemic toxicity [5,6]. However, even with this advance in systemic treatment, the rate of toxicities due to the bystander effect and specific resistance mechanisms can lead to cancer refractoriness.

The resistance mechanisms of the tumor to therapies as well as the frailty of some patients are a significant topic in oncology. An intriguing field of research focuses on the way to overcome the resistance to different agents. The intratumoral approach seems to be a valuable way to control the disease in fragile patients.

In the following literature review, we report different loco-regional treatments that may play a role in the future management of NSCLC, as well as discussing their role in different stages of disease and discussing future uses that may synergize with current therapeutic scenarios, thereby increasing their efficacy.

## 2. Intratumoral Therapy: Biological Principles

The evidence that supports the delivery of the therapy directly into the tumor is a very valuable discovery. The main purpose of this approach is to inject a higher dosage of chemotherapy to obtain a better response with fewer adverse effects according to the log-kill hypothesis. Furthermore, it has also been observed that efficacy can be improved with local delivery of intratumoral drugs. In fact, intratumoral treatment can act as a type of carrier, including as a passive nanocarrier of actively targeted therapeutics (e.g., AdCs) or cells themselves [7]. However, data show that in order to obtain better control the disease, it is imperative to modify the tumor microenvironment, thus increasing the overall efficacy of the therapy and improving its potency [8,9].

This microenvironment manipulation can be observed with immunotherapy when a tumor switches from “cold” to “hot”, a process caused by the infiltration of a high number of cytotoxic T lymphocytes; such a modification is associated with an improved prognosis. “Cold” tumors show poor immune infiltration (specifically the absence of T cells infiltrates) and are typically linked to a poorer prognosis.

Another way to condition the tumoral microenvironment is the use of SBRT (stereotactic body radiation therapy), which can “turn” tumors from “cold” to “hot” [10]. The combination of SBRT with PD-1/PD-L1 inhibitors creates a synergistic effect by enhancing positive immunoregulation and attenuating negative immune resistance. PD-1/PD-L1 inhibitors can attenuate radio resistance and boost abscopal effects [11,12].

The principle of intratumoral therapy is to improve not only the local activity but also the systemic one. For example, intratumoral injection of immunotherapy or chemotherapy may have a favorable antitumor immunoresponse because, by modifying the TME, it can stimulate the immune system through the production of antigens, which create specific antibodies. In this way, it can prime or boost the local antitumor immune response and generate a systemic response against non-injected lesions through circulating activated immune cells. In addition, this immunomodulation may be superior to systemic therapy (e.g., immunotherapy) because it is devised from the heterogeneity of a particular tumor [13,14,15]. Despite this, a robust immune system is a necessary condition, so that the antitumor immune response is effective [16,17]; this robust immune system may be invalidated totally or partially by systemic therapies (e.g., chemotherapy).

To date, several intratumoral therapies have been developed that are aimed at addressing specific conditions; among these are locoregional drugs/chemotherapy (including inhaled versions), brachytherapy, photodynamic therapy, radiation therapy, and thermal or non-thermal local ablative methods.

Intratumoral therapies may have a wide range of applications, including in the neoadjuvant setting/early stage [18], as well as in the metastatic setting. This latter setting, especially in patients with oligometastatic disease, may represent a good field in which to develop intratumoral therapies.

In 1995, Hellman and Weichselbaum proposed “the existence of a clinical significant state of oligometastases” [19] where oligometastases were initially defined by a “limited” number of clinically detectable metastatic lesions. They hypothesized a transition state between localized and widespread systemic disease. Moreover, they proposed that local control of oligometastases could delay, or even avoid, systemic spreading.

Although the definition of oligometastatic disease (OMD) is variable, it is commonly defined as a maximum of five metastases in up to three different organs. During the NSCLC patient journey, an oligometastatic state occurs in up to 50% of cases [20,21,22]. Imaging systems such as Nuclear Magnetic Resonance (NMR), Computed Tomography (CT) scan, PET FDG, and other functional image-based on biomarker tracers are fundamental when seeking to accurately detect every lesion with high-definition details of key tumor features [23,24].

Oligometastatic disease can be synchronous if it is discovered simultaneously with the diagnosis of the primary tumor. The term metachronous is used if the OMD is discovered between 2–3 months after the initial diagnosis according to the definition of Niibe and Hayakawa [25]. By definition, the state of oligoprogression occurs after initially successful treatment of metastatic disease and where disease progression is encountered only in a minority of the affected sites. Finally, the state of oligopersistence is defined as the point when the disease remains stable or shows a partial positive response to systemic therapy [26].

A biological definition of OMD is still lacking and biomolecular signatures of this tumoral behaviour are not completely understood. However, a particular OMD genomic profile might help to predict this status, thereby improving the possibility of specific therapeutic strategies, such as local, systemic, or combination strategies [27].

This paper reviews some intratumoral therapies, in particular treatments that literally “collide” with the tumor and its microscopical environment, e.g., such as the intratumoral injection of chemo- and immunotherapy, photodynamic therapy, as well as an emerging technology known as nanoparticle therapy. In this paper, we try to shed light on the potential role of intratumoral therapeutic advances in NSCLC treatment and their possible application in the management of oligometastatic, oligoprogressive, and locally advanced disease conditions.

## 3. Research Methods

PUBMED, EMBASE, Cochrane Library, and clinicaltrials.gov databases have been used for our review. We have chosen them as they provide robust scientific data, in particular phase I, II, and III phase trials, as well as comprehensive reviews and relevant clinical and preclinical research data. Articles published in the English language between 1 January 1995 and 1 August 2024 were selected. Finally, our research has evaluated some “key words”: “intratumoral treatment” OR “intratumoral injection” AND “intratumoral chemotherapy” AND “local immunotherapy treatment” OR “intratumoral immunotherapy”, “lung cancer oligometastasis local treatment” AND “NSCLC oligometastasis”, “nanoparticle intratumoral delivery” AND “radioenhancers local treatment”.

## 4. Results

### 4.1. Delivery of Effective Intratumoral Treatment

A large amount of the literature published up until today is focused on those intratumoral therapies that encompass antiblastic chemotherapeutic agents and immunocheck-point inhibitors.

#### 4.1.1. Intratumoral Antiblastic Strategy

Cisplatin alone or in combination with immunotherapy was the drug used in the first trials in a palliative setting.

In 2013, Hohenforst-Schmidt et al. studied the intratumoral injection of cisplatin in five NSCLC patients with EGFR negative and stage IIIa-IV (1 IIIa, 2 IIIb, 2 IV and out of protocol 1 mammarian IV) cancer. The patients were ECOG PS 2 and were poor candidates for standard oncologic procedures (surgery, radiation, and chemotherapy) with acute local problems (bleeding, atelectasis, stenosis). The study’s protocol consisted of intravenous chemotherapy every four weeks and intratumoral chemotherapy (ITC) every week. There were no severe adverse effects found in a total of 22 ITC sessions. This trial, for the first time, directly treated the involved lymph nodes simultaneously with the tumor mass [16].

In a subsequent effort, intratumoral cisplatin was analyzed in a second line setting. Zarogoulidis et al. recruited 74 patients with NSCLC, PD-L1 < or equal to 50%, only adenocarcinoma or squamous cell carcinoma, and all negative for EGFR, ALK, TK1, and BRAF. All the patients received chemotherapy as their first line treatment, and were separated into 2 groups: 24 patients received intravenous nivolumab or pembrolizumab, while 50 patients received 1/3 of normal dosage of cisplatin and immunotherapy (pembrolizumab or nivolumab). This intratumoral injection was delivered via bronchoscopy, and the remaining doses of both therapies were administered intravenously. Patients’ performance status and disease progress were monitored at 0, 4, and 8 months after intravenous administration. The results showed the absence of severe adverse events and that the best outcome of disease progression and performance status was linked to PD-L1 < or equal to 25%, tumor size < or equal 6 cm, and less than 5 metastasis sites [28].

Even patients in neoadjuvant settings may take advantage of intratumoral chemotherapy. In 2021, a phase 1 trial (NCT04809103) started; the study is ongoing and is analyzing the use of intratumoral cisplatin in resectable NSCLC. The theory that supports the approach taken by the trial is compelling because neoadjuvant intratumoral therapy could achieve immune priming and may reduce the possible risk of failure of immunotherapy, which is typically caused by lack of presentation of tumor antigens that can be targeted by the immune system.

The primary objective is to find the balance between the dose’s efficacy and its toxicity. There are several key metrics that the testing is attempting to establish. This trial predicts a major tumor response at surgical resection following 30 days of bronchoscopic therapy. Additional metrics include blood-based biomarkers such as cytokine analysis, mass cytometry for inflammatory cells, and a complete blood count, as well as a tissue biomarker panel taken at bronchoscopy and at surgery [29].

Cisplatin was studied in combination with endostar by Ji et al. Endostar is a recombinant human endostatin with a significant anti-tumor angiogenesis effect that functions by reducing the expression of VEGF. Thus, this agent can inhibit tumor neovascularization, promote cancer cell apoptosis, and inhibit tumor infiltration and metastasis. The study consisted of 40 patients with lung squamous cell carcinoma—4 IIIb and 36 IV stage—who underwent conventional chemotherapy +/− radiotherapy over a period of 21 days. Twenty patients received an intratumoral injection of cisplatin (20 mg) and endostar (15 mg) via bronchoscopy on the third and tenth day of each cycle. The intratumoral group experienced greater short- and long-term therapeutic efficacy compared to the control group. In general, adverse effects were similar between the two groups, even when adverse cardiovascular reactions were observed in the intratumoral group; there was also one case of ST-T change and two cases of blood pressure fluctuations, most likely caused by endostar [30].

Finally, the potential use of para-toluenesulfonamide (PTS), which belongs to sulfonamide group and is a low molecular weight organic compound with anti-tumoral activity, by injecting intratumorally should be noted. It may induce tumor cell death in an apparently quite selective way because it causes mild injury in the near normal tissue [31,32].

This antiblastic agent was employed in a phase III trial that analyzed palliative injections in 90 NSCLC patients with severe malignant airway obstruction. The authors showed both the efficacy of the treatment as well as its good safety profile [33].

#### 4.1.2. Intratumoral Immunomodulation

Immunotherapy, with its antitumoral role, has revolutionized the toolset available in oncology. One example is the enhancement of T-cell responses by taking advantage of the full repertoire of available tumor antigens. According to Yang et al., in murine models, chemokine CCL21 treatment increased CD4, CD8, and CD11c+DEC205+ DC infiltration into the tumor, which caused its microenvironment to acquire a lymphoid-like aspect [34]. Dendritic cells (DC) also play an important role in immune activity modulation by presenting cells to stimulate naïve T cells in the immune system.

While the first DC-based cancer immunotherapy for prostate metastatic cancer [35] approved by the FDA was developed in 2010, in the past few years, research has turned towards finding a more efficient and robust method of DC stimulation and maturation. This has been achieved by using autologous DC to overexpress CCL21, which was the basis of a phase I trial [36] that delivered an injection of autologous DC that overexpressed CCL21 (AdCCL21-DC) in advanced NSCLC.

A cohort of 16 stage IIIB/IV NSCLC patients received 2 vaccinations (1 × 106, 5 × 106, 1 × 107, or 3 × 107 DCs/injection) by CT- or bronchoscopic-guided intratumoral injections (days 0 and 7). The disease in 4 patients (25%) became stable on the 56th day after injection, with a median survival of 3.9 months. The typical adverse events were fatigue, dyspnoea, and pain/muscle weakness, and there appears to be no clear association with dose or schedule. Patient response was monitored by ELISPOT assays, which detected six systemic responses against tumor-associated antigens (TAA). Tumor CD8+ T-cell infiltration was induced in 54% of subjects (7/13), with a range between 1.3 and 7.7 range and a 3.4 fold average increase. Accordingly, patients with increased CD8+ T cells showed significantly increased PD-L1 mRNA expression, which provides a benefit in PD-1/PD-L1 checkpoint inhibition. This may be further enhanced by vaccination for all cases, including low PD-L1 baseline-expressing tumors and those that show a paucity of CD8+ T-cell infiltration [36].

A current and ongoing phase I trial is investigating the potential synergy between checkpoint inhibitors and CCL21-DC for stage IV NSCLC patients [37]. The trial’s primary endpoint is defining the maximum tolerated dose and the dose expansion. Secondary endpoints are the detection of adverse events and drug target activity, especially through immunological monitoring.

Another interesting trial [38] was a randomized phase II study investigating SBRT and systemic pembrolizumab with or without intratumoral avelumab/ipilimumab plus CD1c (BDCA-1) +/CD141 (BDCA-3) + myeloid dendritic cells in oligoprogressive solid tumors, also NSCLC, refractory to anti PD-1 ICB. Myeloid dendritic cells—in particular CD1c(BDCA-1)—play a pivotal role in favorable and “hot” tumor microenvironments. Some preliminary data were published in November 2023 that showed that the intratumoral injection of myDC plus mAb and intravenous pembrolizumab was feasible and tolerable, with early evidence of activity in refractory cancer [39].

A phase II trial [40] combined SBRT with intratumoral oncolytic virus therapy before pembrolizumab in 57 patients with metastatic NSCLC (29 patients) and triple negative breast cancer (28 patients). This oncolytic virus consisted of adenovirus-mediated expression of herpes simplex virus thymidine kinase (ADV/HSV-tk) plus valacyclovir therapy. ADV/HSV-tk was injected intratumorally on Day 0, with administration of valacyclovir from Day 1 to Day 15. Concurrent with this, SBRT of 30 Gy (6 Gy in 5 fractions) was administered from Day 2 to Day 16. Finally, pembrolizumab (200 mg) was administered every 3 weeks starting on Day 17 and continuing until disease progression or toxicity levels became unacceptable or up to 24 months in patients without disease progression. The primary outcome was objective response rate (ORR). The secondary outcomes were duration of response (30 days after the last dose of pembrolizumab until disease progression); overall survival rate; progression-free survival rate; number of treatment-related adverse events; antitumor activity; and clinical benefit rate. In total, 28 NSCLC patients were enrolled, and the preliminary results showed a high tolerance and promising news regarding benefit and response [41].

Another study used RP1, a modified virus (herpes simplex 1) that is designed to directly destroy tumors and to generate an immune response against tumors according to the results of a phase 1–2 trial [42]. The aim was the evaluation of safety and tolerability, biodistribution, and shedding and preliminary efficacy of RP1 in monotherapy and in combination with nivolumab in advanced and/or refractory cancer, including NSCLC. The study is currently ongoing (Table 1).

#### 4.1.3. Intrapleural Therapy

Pleural metastatic spread is a high frequence localization of lung cancer, in particular adenocarcinoma. Often, it is diagnosed before the primary tumor because it can arouse effusion and consequently cause symptoms. Effusion, which is a negative prognostic factor, may require some invasive procedures for the effective management of symptoms (evacuation, pleurodesis etc.). Unfortunately, these maneuvers may not be feasible, for example because a patient is not an optimal surgery candidate or because the effusion is refractory to evacuation. Therefore, it may be useful as a potential intrapleural strategy. Moreover, if the pleura is involved at a single metastatic site, this local approach may be an example of intratumoral therapy in an oligometastatic setting.

Hyperthermic intrathoracic chemotherapy (HITHOC) takes its inspiration from the intraperitoneal procedure. Nowadays, HITHOC may be used in mesothelioma and thymoma [52,53,54] or in secondary pleural metastasis, in particular pseudomyxoma peritonei and ovarian cancer [55,56]. Data on NSCLC are scarce and therapeutic support for IV stage NSCLC is not recommended without proof of benefit [57].

In 2023, a single-center retrospective study [58] compared the efficacy of HITHOC and pleural catheter drainage (IPCD) for initially diagnosed lung cancer patients with symptomatic malignant pleural effusion. In total, 33 patients were evaluated; 10 patients received IPCD and 23 patients underwent HITHOC. This study showed a malignant pleural effusion (MPE) control rate in the IPCD group similar to that of the HITHOC group in the first month. The IPCD group rate was worse than HITHOC in the third month (30% vs. 69.6%). Moreover, the study showed a median time to MPE recurrence in the IPCD group that was shorter than that for the HITHOC group (2.5 months vs. 22.9 months). Although TKIs and chemotherapy were independent protective factors for recurrent MPE, HITHOC was not an independent factor for MPE iPFS and OS in multivariate analysis; only certain subgroups of the TKIs treatment group seemed to have significantly longer iPFS and OS than those treated with IPDC. The chemotherapy group did not show a significant difference in OS between the two treatments; however, the trend seemed to favor the HITHOC group. Finally, the chemotherapy group did not show a better control of MPE, despite the small sample size.

A phase I trial by Adusumilli et al. investigated HITHOC treatment of NSCLC pleural metastasis, based on the new frontier of cohesion between vaccines and ICIs. This trial enrolled 25 malignant pleural mesothelioma patients, 1 breast cancer patient, and 1 NSCLC patient and provided intrapleural administration of mesothelin-targeted chimeric antigen receptor (CAR) T cell therapy, which was shown to be safe and tolerable [59].

#### 4.1.4. Inhalation Strategy

A particular method of intratumoral treatment is inhalation. Its first target site is lung tumoral tissue, with the enrichment of micrometastasis, or tumoral cells in lymph nodes, thanks to lymphatic drainage near the alveoli, which are the gateways for systemic circulation. However, this method has some limitations. First, lung tissue shows some local cell structures and cells (beating cilia, mucus, macrophages, enzymes) that may modify the absorption of inhaled drugs. This is especially relevant in cases of COPD (chronic obstructive pulmonary disease) or other lung pathologies that reduce the inhalation capacity itself or cause an increased drug concentration and consequently cause a non-specific allergic reaction (e.g., bullous emphysema or extended bronchiectasis). Aerosol devices, such as ultrasonic nebulizers and jet nebulizers, do not need coordination between patients and administration, which is a significant advantage. With aerosols, a large quantity of drugs may be lost in the surrounding environment, so the dose must be nebulized in a controlled environment (medical context) as it requires aspiration with special filters.

Some chemotherapies have been studied and designed for delivery via inhalation, in vitro and in vivo, using particular devices, special cages, and/or chambers.

5 fluorouracil (5-FU) and doxorubicin may be coated with lipids or liposome nanoparticles to reduce side effects. In particular, doxorubicin may cause chest pain, cardiac toxicity, alveolar hemorrhage, and wheezing [60,61,62].

Taxanes with cyclosporin A have only been studied in animal settings [63]. Gemcitabine [64,65], 9-nitrocamptothecine (9-NC), and bevacizumab have been investigated for NSCLC. [66,67]. Finally, platinum has been developed in the form of a dry powder to ensure that the nebulized forms are bypassed. Moreover, the dry powder form sustains the release and retention of cisplatin in the lung, increasing drug exposure at the targeted tumor site, rather than through intravenous and nebulization applications [68,69,70].

Inhaled dry powder distributes a smaller dose of chemotherapy and intensifies its administration. As such, CIS-DPI would be a candidate for a metronomic-like schedule. Metronomic cisplatin seems to show anti-angiogenic effects in vivo, with tumor growth reduction [69]. Furthermore, in NSCLC, it may show lower toxicity and higher tolerability. Although the metronomic schedule is promising, the existing data do not yet support a valid comparison with conventional chemotherapy [71,72]. A metronomic-like schedule would reduce systemic exposure and immunosuppression and its activity is favorable for an immune-related antitumor response [68,73]. According to Fournel’s study, cisplatin seems to activate PD-L1 upregulation [74], so cisplatin combined with anti-PD1 may enhance the anti-tumor immune response. Therefore, the combination with ICIs may reduce tumor growth and enhance survival rates over time more than the combination with anti-PD1 monotherapy. However, this interesting therapeutic approach needs to be tested and confirmed [70].

Lung function tests (spirometry, 6-min walking test, and DLCO) and imaging techniques (high-definition TC) must be applied first in order to detect those pathologies that represent the main contraindications: stage IV COPD, severe uncontrolled asthma, and bullous emphysema or extended bronchiectasis [75].

### 4.2. Ablation Strategies

Ablation is a heterogeneous technique because it can take advantage of specific qualities of various physical phenomena, like heat, cold, or light. These define a physiological alteration of cells and the tumor microenvironment with necrosis as the final result.

#### 4.2.1. Photodynamic Therapy

Photodynamic therapy (PDT) is an ablative technique that acts without using ionizing radiation. PDT consists of a photosensitizer drug excited by appropriate wavelength laser irradiation. The reaction with light produces singlet oxygen, which has anticancer activity by inducing apoptosis, necrosis, and autophagic tumor cell death. Photosensitizer drugs accumulate in the specific tumor sites and the newer ones are increasingly focused on tumor cells, offering an extremely specific and lesion-oriented therapeutic strategy with fewer complications [76,77,78]. Talaporfin is an example of a newer photosensitizer that has higher absorption bands at longer wavelengths and shows increased efficacy in tumors under or over 1 cm without notable variation of efficacy [79]. PDT may be used alone, for example in unresectable early central lung cancer NSCLC, or as a part of a multimodal treatment, for instance in the palliative setting [80].

Unfortunately, PDT cannot be used for deeper lesions because light penetration of tissue is very low. A too dense or necrotic mass may be linked to a lower amount of oxygen and the reduced effectiveness of PDT. Moreover, PDT has a waiting time of photosensitizer administration to laser light illumination that is defined as the “drug-light interval”. This is typically between 24–96 h, although chlorine e6, a vascular-targeted photosensitizer, may reduce the interval time to 3 h [81]. Treatment adverse effects are minimal and temporary, e.g., cough, painful breathing, burning, swelling and scarring in the nearby tissue.

The future of PDT is firmly connected with Nano Particles (NPs). These may improve penetration and deliver cell specific photosensitizers [82] to lung cancers, thus improving the immune effects of PDT. These effects can be further enhanced with other ablative techniques to build a strong connection and empower immunotherapies. An example of this strong synergy is PpIX-1MT [83], a chimeric peptide, that joins photosensitizers PpIX and 1MT—an immune checkpoint—that could efficiently recruit CD8+T cells; this has been demonstrated in both in vitro and in vivo studies. Upon receiving 630 nm light irradiation, these nanoparticles will produce reactive oxygen species, induce apoptosis, and provoke expression of caspase-3. In doing so, they induce the production of tumor antigens and support an immune response. A recent review [84] draws attention to PDT immune stimulation and the strong relationship with immunotherapy, especially based on PD-1/PD-L1 blockade [85]. PDT may be used as a part of a combined therapy (immune or chemotherapy) [86], typified by two different strategies of action.

#### 4.2.2. Thermal Ablation

Thermal ablation is an intratumoral approach that takes advantage of extreme temperatures to induce tissue damage and modify the tumoral microenvironment. We have three different modalities at our disposal that may be delivered percutaneously directly into the tumor (as is the current clinical practice), or by bronchoscopy, which is an emerging and promising methodology (Table 2). Oligometastatic disease may benefit from these techniques. The maximum number of metastases in a single patient that can be treated is not clear, but is typically between 3 and 5. Attempts to address more than 3–5 metastases typically result in a poorer prognosis. A further limiting factor is the size of the metastases; a tumor greater than 2 cm may be associated with worse OS [22]. Thermal ablation is usually a strategy for primary cancer therapies. ESMO and ACCP have selected this method for patients who present contraindications to surgery or stereotactic radiotherapy (cardiorespiratory comorbidity or insufficient vital lung reserve). This approach is effective because a better outcome is linked to first stage tumors that are less than 2 cm [87,88,89,90].

Thermal ablation is generally well tolerated, so it has no absolute contraindication except for the untreatable coagulopathy [91]. Instead, it shows technical contraindications: lesions that are <1 cm from hilium, large vessels, or main bronchi, oesophagus, or trachea, and tumors abutting a vessel > 3 mm or the myocardium. However, a patient with an ECOG PS > 2 or a life expectancy less than 1 year is not a good candidate for lung ablation.

Some recent data show that thermal ablative therapies may improve the efficacy of immunotherapy for cancer patients [92,93]. The evidence has been derived from other malignancies like colorectal, prostate, and melanoma. Tumor necrosis, caused by thermal shock, releases neoantigens, so there is an increase of danger signals (endogenous molecules released by damaged cells) that, together, assist in modulating the first steps of the cancer immunity cycle. The induction of neoantigen expression is linked to neoantigen presentation as evidenced by the elevated number of dendritic cells; this effect was also observed after ablation in NSCLC patients [94,95,96]. Moreover, some studies have shown that after ablation, RFA, cryoablation, and fewer MWA [97,98] lymphocytes (T CD4+ and CD8+) are predominant in the peritumoral infiltration [95,99,100,101]. Further, PD-L1 and PD1 expression are upregulated in tumor infiltrating CD8+ and CD4+. Post thermal ablation seems to have beneficial effects on tumor progression and survival as it results in a higher number of CD4+ and CD8+ and a lower number of Treg cells and myeloid-derived suppressor cells [102,103]. Unfortunately, immune response post thermal ablation is transient [104], but it enhances the effect of immunotherapies by upregulating various steps in the cancer immunity process. Combination treatments are linked to a more sustained immune response than either ICI or thermal ablation alone [105].

In 2013, Yuangying et al. conducted a retrospective study that showed that a combination of cryotherapy with chemotherapy or immunotherapy significantly increased OS compared to single chemo- or immunotherapy. However, the median OS of patients who received cryo-chemo-immunotherapy was significantly longer [106].

In future, NSCLC studies will define the synergy of thermal ablation with ICI [107,108,109,110,111,112,113,114].

Recently, a phase 1b/2 trial [110] evaluated the effect of an intratumoral injection of IP-001 in combination with RFA. IP-001 is a specific variant of the N-dihydrogalactochitosan (GC) molecule family and plays a multi-function role in immune stimulation when used with ablation. The direct effect is to inhibit cell membrane repair and the recycling of ablation-damaged tumor cells, while the indirect effect may sequester ablation-released tumor antigens and induce a potent Th1 type T cell response against the cancer by recruiting and stimulating antigen presenting cells [115].

**Table 2:** Synopsis of different thermal ablation techniques.

Technique of Ablation	Type of Energy	Notes	Complications [87,116,117,118,119,120]
Radiofrequency (RFA)	Alternating current (heat)	Dependent on conductance which is low in lungs.	RFA is the most investigated procedure. PNX is the most common complication (30–67%; grade 3 is <2%) and the rate of tube placement is 13–21%. Overall procedure-related rate of major complications is 9.8% (mainly interstitial pneumonia and haemothorax) and mortality is 0.4%.
Microwave (MWA)	Electromagnetic waves (heat > 100°)	Thanks to higher energy and higher temperature than RFA, the addiction of tissue impedance is reduced as is the heat-sink effect; but the greater energy may cause more potential problems than RFA.	
Cryoablation	Temperature reduction (−160°)	Less painful but it has a lower deed filled –compared to others, so it requires more cryoprobes, with an elevation of complication	

#### 4.2.3. Other Thermal Ablative Strategies

In the last decade, some preclinical studies analyzed other thermal ablative strategies. For example, Bronchoscopic thermal vapor ablation (BTVA-C) has been used in lung volume reduction for emphysema [121]. In 2015, Henne et al. [122], and in 2018, Ferguson et al. [123], showed encouraging results, suggesting the potential of minimally invading ablation of peripheral cancerous lesions. In 2019, a clinical study (Vaporize) commenced. Patients were enrolled if they had primary lung cancer or metastatic cancer in the lung; the patients also had to have undergone scheduled surgical resection. Vaporize shows promise and a high tolerance, in particular for smaller volumes, on the condition that the recommended thermal dose is applied [124,125]. A subsequent study, called Vaporized, focused on BTVA-C in primary lung cancer or metastatic cancer in lung patients who are not recommended for surgery. This trial is ongoing [126].

Finally, laser Interstitial Thermal Therapy, which is another ablative method using a diode laser to induce tissue charring, is under investigation. Casal et al. used the highest power settings and laser delivery fiber on a porcine lung parenchyma [127,128]. BLAST-SR is a currently ongoing trial that evaluates pathologic changes after laser treatment [129].

### 4.3. Brachytherapy

Although this article does not intend to cover the role of RT in the management of NSCLC, for which reference is made to other reviews [88,130,131,132,133,134,135], it seems useful to include brachytherapy among the various treatment options as a possible intratumoral therapy.

High dose rate brachytherapy (HDR-BT) is used primarily in palliative settings [136]. The indications for lung cancer are typically an obstructive disease with several symptoms (dyspnoea, haemoptysis, bronchopneumonia, atelectasis). In such cases, BT can be used in monotherapy or in combination with external beam radiotherapy (EBRT) (massive lymph node involvement) laser resection, implantation of prostheses, and cryotherapy. Other indications include treatment of recurrence after surgery and/or EBRT. The primary advantage of BT, as compared with mechanical techniques (laser ablation, cryosurgery, electrocautery endoscopic biopsy forceps), is the modification of the tumor kinetic and better control of malignant forms. Depending on the location of the lesion, quality of life may be improved quickly by BT within as little as a few hours to, at most, a few days [137]. The dose may vary between 10 to 15 Gy, mainly because there is little empirical evidence regarding the correct value [138]. Moreover, healthy tissue damage is reduced as the dosage frequency falls off [139].

On the other hand, HDR-BT has a limited role in radical settings with curative intent [136,139,140]. This method may be used in selected cases of early endobronchial cancer or after resection with positive margins [140] with or without chemotherapy. For example, when traditional thoracotomy and lobectomy are not available options, a sub-lobar resection with permanent implantation of I125 vycril mesh might be a more feasible option [141,142,143]. This implantation may be used safely in subpleural, peripheral, and Pancost tumors [140], as well as in delicate zones such as in para-spinal tumors with high localized doses to the spinal cord [144]. Youroukou et al. analyzed interstitial BT for IA and IB, showing an excellent local in field control rate despite the lack of clinical information about recurrence rate between sub lobar reception versus sub lobar resection associated brachytherapy mesh [145]. We have some data concerning the comparison between HDR BT and EBRT. According to Patel et al., NSCLC (T1-T4 N0 M0) patients treated with limited resection showed similar survival rates between the HDR BT group and EBRT group, and survival was better in patients treated with HDR BT [143] (Table 3).

### 4.4. Tumor Treating Fields (TTFields)

In the last two decades there has been an increased interest in the use of electric fields in oncological therapies. [147,148]. Alternating electric fields with low-intensity and intermediate frequency (100–300 kHz) have been shown to inhibit the growth of tumor cell across various cell lines. This very intriguing capability has given rise to the use of tumor treating fields (TTF) [149,150]. In a phase I/II clinical trial, for the first time, TTF (NovoTTF-100L), in association with pemetrexed, played a role in locally advanced and pretreated NSCLC (NCT00749346) [151]. The phase I trial showed a good tolerance without serious AEs. The phase II trial showed an improvement in disease control and increased treatment efficacy [152].

In 2016, a phase III trial (NCT02973789) [153] was designed to test the efficacy and safety of TTFields (NovoTTF-200T device) for stage IV NSCLC (squamous or no squamous) during or after a platinum-based treatment. TTFields were associated with immune checkpoint inhibitors or docetaxel (standard of care) and compared with standard of care alone. The results show a significant improvement of OS with TTfields therapy without enhancing systemic toxicities; its safety profile presented mainly low-grade cutaneous toxicity, and there were no related deaths.

The LUNAR study is the first randomized, pivotal phase III study to examine TTFields therapy for NSCLC. The study aim was to help physicians to face the unmet need for new options that can improve survival in second-line therapy and beyond for patients with metastatic NSCLC.

Before LUNAR, and since the OAK study of atezolizumab in 2017 (NCT02008227), no phase III study that includes patients without driver mutations had shown an OS improvement after progression compared to platinum-based therapy.

In the study, the addition of TTFields therapy significantly improved OS when compared to an immune checkpoint inhibitor alone, with respective median OS of 18.5 months and 10.8 months. TTF with immunotherapy subgroups shows better results than the TTF with docetaxel group [154]. TTFields therapy must be considered as an innovative first-in-class treatment method that may be integrated into clinical practice and added to existing treatments.

LUNAR2 (NCT06216301) commenced in January 2024 [155]. This is a phase III trial that will investigate the effectiveness and safety of TTFields (NovoTTF-200T device) combined with the administration of pembrolizumab and platinum-based chemotherapy in patients with stage IV NSCLC with untreated metastasis versus pembrolizumab and platinum-based chemotherapy alone.

### 4.5. Nanoparticles

During the last decade, an emerging interest in nanomedicine and in nanotechnological applications has steadily gained ground. This has been developed to address issues such as drug resistance, low drug efficacy, and side effects. Nanoparticles (NPs) have a very high surface-to-volume ratio as a result of their nanoscale dimensions [156]. In this way, multiple ligands may attach to the surface, which in turn may develop multiple covalent bonds. Thus, nanoscale platforms allow for better treatment efficacy and accuracy in terms of localizing drug delivery. Moreover, nanoarchitectures could encapsulate different drugs, especially hydrophobic drugs.

NPs have been studied for their potential anti-inflammatory, antioxidant, and immunomodulatory effects. Suppression of proinflammatory mediators (IL-6, IL-8, IL-1beta, and TNF-alpha) and deactivation of CD4 and CD8 cells in lung tissue [157] has also been observed. NPs could be linked to induced ROS, leading to apoptosis and necrosis [158]. In addition, they can downregulate Toll-like receptor 4, which is associated with carcinogenesis [159], VEGF, HIF-1alpha, IL8, and Bcl-2 genes. They can also increase the gene expression of p53 [160]. Moreover, the tumor’s capability to evade apoptosis could be a target for NPs, as Akbarzadeh revealed in 2022, albeit in the treatment of breast cancer. For example, magnetic γ-Fe2O3 nanoparticles (MNPs) associated with cationic poly-l-lysine (PLL) could be used to construct the PLL-MNPs. [161]. Further, necrosis could be targeted, inducing dysfunction of the endothelial cell (via the von Willebrand factor), or, thanks to formation of ROS, inflammatory cytokines and/or activation of the coagulation system. An example may be gold nanoparticles, which can enhance ROS production, sensitize mitochondrial membrane potential, and stimulate both primary and late apoptosis in lung cancer by binding to various functional groups. These versatile features could benefit gene therapy, drug delivery, photothermal therapy, and photodynamic therapy, in both viral diseases and in lung cancers [162,163].

Nanoparticles may merge with traditional therapeutic strategies such as chemotherapy, immunotherapy, and radiotherapy, as well as non-conventional elements.

The best-known chemotherapy compound is nab-paclitaxel, which is a nanoparticle albumin-bound paclitaxel. This compound has demonstrated clinical efficacy in the treatment of advanced NSCLC either as monotherapy or in combination [164]. According to preliminary results from the Keynote-407 and IMpower 131 studies, it may be possible to improve clinical benefits with manageable toxicity by using novel combinations of nab-paclitaxel/carboplatin and PD-1/PD-L1 inhibitors. [165,166]. Recently, HLX10-004-NSCLC303, a randomized, double-blind, multicentre, phase III clinical study has aimed to compare the clinical efficacy and safety of HLX10 + chemotherapy vs. chemotherapy alone in subjects with locally advanced or metastatic squamous NSCLC who have not previously received systemic treatment. Chemotherapy is based on Carboplatin Nanoparticle Albumin Bound (Nab)-Paclitaxel [167]. Paclitaxel may be also transformed into submicron particles and, according to the NCT04314895 trial, it has been evaluated for injection directly into lung cancer patients [168]. Cisplatin (CDDP) has been studied as a possible drug that can be carried by NPS. In 2019, Sun et al. created a liposomal PEITC-CDDP. CDDP was encapsulated in a liposomal NP with phenethyl isothiocyanate (PEITC), which may sensibilize lung cancer to CDDP. This compound showed an increased time of circulation with lower side-effects; this is because it produces toxicity mainly toward NSCLC cells, with good sparing of normal cells [169]. Gefitinib is also engaged in nanoparticles encapsulation and it has been analyzed as part of a compound with chitosan, cyclodextrin, liposomes, poly (lactic-co-glycolic acid), polylactide, solid lipid nanoparticles, nanostructured lipid carriers, and albumin nanoparticles. Compounds with nanoliposomes show better properties because they may increase stability and promote proapoptotic activity on A549 cells, thereby enhancing gefitinib activities, for example inhibition of proliferation, migration, and invasion of tumor cells [170,171].

Quaratusugene ozeplasmid consists of non-viral lipid nanoparticles that encapsulate a DNA plasmid containing TUSC2, a tumor suppressor gene that is decreased in 82% of patients with NSCLC. There are currently two ongoing studies focused on quaratusugene ozeplasmid: Acclaim-1 and Acclaim-2. Acclaim 1 is a randomized study that aims to determine the safety and efficacy of quaratusugene ezeplasmid added to Osimertinib in NSCLC patients with mutated EGFR and who have progressed with Osimertinib [172,173]. Acclaim-2 is a Phase I–II multicenter open-label study. NPs will be used in combination with pembrolizumab in patients with locally advanced or metastatic NSCLC with any PD-L1 TPS; the patient is eligible if they have shown almost three months of clinical benefit pursuant to a pembrolizumab-based treatment.

In 2019, brachytherapy was integrated with NPs with the creation of “188Re-ImDendrim”, a fifth generation poly-L-lysine dendrimer plus [188Re]-rhenium. This study followed the administration directly into five patients’ lung tumors under CT-guidance. The initial data were promising concerning safety and tolerance [174].

NPs may grant non-“conventional” elements a potential therapeutic role. These elements must have an anti-inflammatory or antitumor potential in vitro and in vivo. Curcumin nanoparticles (Cur-NPS) in vitro induced apoptosis and caused G2/M arrest in both A549 and Calu-3 cell lines during a study on inhalation [175]. Zerumbone (ZER) and Berberine-phytantriol (BP) may be delivered with higher efficiency by liquid crystalline nanoparticles (LCNs and BP-LCNs). Both may inhibit the proliferation and migration of A549 cells by regulating tumor suppressor genes P53 and PTEN and metastasis-associated gene KRT18 [176,177]. MiRNAs are responsible for post-transcriptional control of gene expression and are frequently downregulated in cancer. Preclinical evidence shows that restoring miRNA levels can inhibit tumor growth. MiRNA may be delivered by EDVTM nano cells (EDVs) that are bacterially derived (Targomir). This therapeutic strategy had been studied in a Phase I clinical trial (Mesomir-1) with a promising outcome, even if initial results seemed to show that MiRNA therapy might not be effective in all patients [178].

## 5. Discussion

In this review, the possibilities of using intratumoral strategies (summarized in Table 4) to offer efficient therapy with a related decrease in adverse events have been evaluated. It is possible to take advantage of various strategies according to qualities of both cancer and patient.

“Traditional” antiblastic therapy has been analyzed not only in the palliative setting but also in the neoadjuvant setting and in frail patients, too [16]. The use of intratumoral therapy in the neoadjuvant setting is a very promising field of research since it could enhance systemic immunotherapy by enhancing the presentation of tumor antigens [29]. As a result, better control of the disease and an improvement in overall survival is hopefully achievable. Considering all the intratumoral strategies described in this review, intratumoral immunomodulation appears to be the most promising based on its tolerability and efficacy data in refractory cancer [39]. However, both strategies require confirmatory data and robust evidence from clinical randomized trials.

In addition, these strategies can be grouped with other therapeutic strategies, including SBRT and systemic immunotherapy, both of which show promising evidence of tolerance and efficacy [39,40]. If intrapleural administration does not offer robust and harmonious evidence because it requires systematic trials, the inhalation strategy offers better evidence, though mainly derived from preclinical studies.

Ablation strategies provide for the use of physical phenomena to destabilize the cancer architecture and to provoke necrosis of tumoral cells. It is important to develop and consider the close relationship with immunotherapy [106] and chemotherapy [86,106] and their possible enhancement.

HDR-BT can be a valid and useful intratumoral strategy in palliative settings [136], with a fast improvement in quality of life [137]. In a radical setting, it has strict indications [140,144].

TTFields are a promising technique with a significant efficacy and an excellent safety profile. Unfortunately, the device is quite expensive and may impact the daily life of patients, because the device must be worn for a long period.

NPs are a real indicator of the future of intratumoral therapy. They have been studied for their potential anti-inflammatory, antioxidant, and immunomodulant roles. Moreover, they are carriers, able to transport anti-tumor drugs with better efficiency, thereby permitting the use of those drugs that cannot be employed alone. In addition, they can play a cross functional role with other intratumoral strategies. According to their versatility, NPs may be a promising technology; however, additional experimentation and clinical studies are necessary to truly understand their efficacy.

A really interesting narrative thread is not only the combination between intratumoral therapies but also the combination between systemic and intratumoral therapies. For example, intratumoral strategies show an increased immunity against tumors, so a combination with immunotherapy can be a powerful strategy that acts against the mechanisms of resistance. This symbiotic therapy might be used in the neoadjuvant setting, since the immune response is stronger and is tailored to suit the tumor. The combination with immunotherapy could enhance the efficacy of the treatments and reduce the risk of future relapse.

Unfortunately, the deficiency of phase II and phase III trials dedicated to intratumoral strategies is not trivial. This lack is a limitation for our review, because we must often draw fully from preclinical studies and phase I trials.

## 6. Conclusions

Nowadays, a key target in the antitumor “war” is the “tumoral sanctuary”, the real kernel of a cancer that consists of drug resistance cells and niches of cancer stem cells. The nature of the tumor (abnormal and chaotic growth of vessel structure etc.) and the tumoral microenvironment protects the “sanctuary” from the “internal” protection system (e.g., immune system) and “external” therapy (e.g., chemo- or immunotherapy). Moreover, cancer paradoxically takes advantage of the “external”/systemic therapy for selecting resistance cells.

As such, we should seek to pierce the “cancer armour” in order to enter into the “cancer sanctuary”. The intratumoral strategy can be a good starting point based to its significant versatility and flexibility to various likelihoods and settings.

An intratumoral strategy has both a local domain and a systemic one. In fact, a direct action on tissues and lymph nodes near the tumor and a systemic diffusion of intratumoral injected chemotherapy and/or immunotherapy by hematic and lymphatic system can be observed.

Moreover, intratumoral strategies may act as synergetic therapies, enhancing the efficacy of the systemic strategy. We are still at the beginning of this road, but we are confident it may be a winning one, even if we need confirmation from in vivo studies. We hope this review, despite its limitations, can be useful as an inspiration.

## Figures and Tables

**Table 1:** Clinical trials concerning intratumoral therapies.

Trial	Intratumoral Therapy	Principle	Strategy	Notes
NCT01574222 phase I [36]	autologous DC overexpressing CCL21 (AdCCL21-DC)	Chemokines CCL21 treatment increased CD4, CD8, and CD11c+DEC205+ DC infiltrates into the tumor, which of microenvironment acquired a lymphoid-like aspect. Also, dendritic cells (DC) have an important role of immune activity modulation, because of being potent antigen by presenting cells to stimulate naïve T cells in immune system.	Intratumoral vaccination with AdCCL21-DC	4 patients (25%) showed stable disease at 56th day after injection, with a median survival of 3.9 months. 4 cases of AEs According to ELISPOT assays, 6 systemic responses against tumor-associated antigens (TAA). Tumor CD8+ T-cell infiltration was induced in 54% of subjects (7/13)
NCT03546361 phase I [37]			Potential synergy between checkpoint inhibitors (pembrolizumab) and CCL21-DC.	Maximum tolerated dose (MTD)/maximum administered dose (MAD) (dose escalation) at 28 days, and overall response rate (ORR) up to 1 year.
NCT04571632 [38] phase II	CD1c (BDCA-1)+/CD141 (BDCA-3)+ myeloid dendritic cells	CD1c(BDCA-1)- have a pivotal role for a favorable and “hot” tumor microenvironment	SBRT with systemic pembrolizumab with or without intratumoral avelumab/ipilimumab plus CD1c (BDCA-1)+/CD141 (BDCA-3)+ myeloid dendritic cells	In Nov. 2023 some preliminary data, in particular the intratumoral injection of myDC plus mAb and iv. pembrolizumab was feasible and tolerable, with early evidence of activity in refractory cancer [38].
NCT03004183 phase II [40]	An oncolytic virus which is adenovirus-mediated expression of herpes simplex virus thymidine kinase (ADV/HSV-tk)	HSV-tk may induce immune-related activities through necrosis mediated exposure of putative tumor antigens to the cytokine-stimulated lymphocytic infiltrate	SBRT with Intratumoral oncolytic virus therapy (plus valacyclovir), as therapeutic opportunity before pembrolizumab.	objective response rate (ORR). 28 NSCLC patients were enrolled, the preliminary results showed a high tolerance and promising news about benefit and response.
NCT03767348 Phase I-II [41]	RP1 a modified virus (herpes simplex 1)	is designed to directly destroy tumors and to generate an immune response against tumors	RP1 in monotherapy and in combination with nivolumab in advanced and/or refractory cancer	Percentage of AEs and severe AEs. Percentage of dose limiting toxicities. Percentage of ORR. Maximum tolerated dose
NCT05265650 [43] phase Ib/II	BO-112 nanoplexed form of poly I:C based viral mimetic [44]	BO-112 may revert anti-PD-1 resistance [45]	Combination with anti-PD-1 mAb (nivolumab) and SABR.	Incidence of AEs and estimation of severe AEs
NCT05076760 [46] phase I	MEM-288 conditionally-replicative oncolytic adenovirus expressing human IFNβ and a recombinant membrane-stable form of CD40L (MEM40)	inhibiting abscopal tumor growth in monotherapy. Synergy with immune checkpoint inhibitors (ICI)	Monotherapy or combination with nivolumab	In Jan. 2023, preliminary data are encouraging about safety, antitumor and immune response (monotherapy arm) [47]
NCT05602792 [48] phase I/IIa	T3011 herpes virus (oncolytic herpes virus) is an attenuated virus which is inserted biologically active IL-12 and anti-PD-1 antibody genes in	T3011 improves TEM and exalt the body’s specific anti-tumor immunity. while lysing tumor cells	monotherapy	In Jan. 2023, preliminary data showed excellent safety profile and encouraging anti-tumor activity (in neck tumors) [49]
NCT04370587 [50] phase I/IIa			Monotherapy or In combination with IV pembrolizumab	(Preliminary data in melanoma) monotherapy and combination were safe and tolerable with encouraging data about efficacy in immune resistant melanoma, with the suggestion of microenvriomental change and bypassing immune resistance [51].

**Table 3:** Overview of brachytherapy in NSCLC.

Contraindications	Common Adverse Events [136]	Risk Factors
- peripheral tumor location and Pancoast tumor (both cases may be treated with interstitial BT) - external pressure (e.g., lymph node compression) - contraindications for bronchoscopy	- PNX and bronchospasm; - hemoptysis (most linked to tumor progression; - pneumonia; - cardiac arrhythmia/arrest - hypotension - necrosis (late complication). - trachea-esophageal fistula linked to infiltration of esophageal wall [146].	- high dose of EBRT - several brachytherapy fractions - tumoral localization in the left upper lobe - long sections of irradiated bronchi.

**Table 4:** Summaries of the main characteristics of the described intratumoral strategies.

Intratumoral Strategy	Description	Advantages	Disadvantages/Limitations
Injection of antiblastic therapy	“Traditional”chemotherapy is administered directly in the tumor mainly by bronchoscopy. It is possible to find the association with intravenous chemotherapy and/or immunotherapy, radiotherapy. The only III phase trial has analyzed the use of PTS in monotherapy [33].	The direct administration may empower the efficacy not only locally but also in the systemic panorama, because intratumoral may have a synergic activity with concomitant systemic therapy or subsequent one, for example by reducing the risk of immunotherapy failure. Moreover, precise therapy seems to have a good safety profile.	The scientific literature returns only one phase III trial, where an intratumoral therapy is administered alone [33]. This treatment is often used in palliative setting. Finally, this treatment is strictly linked to bronchoscopy, a quite invasive exam, with its particular complications and adverse events.
Injection of immunomodulant	immunomodulant is administered directly in the tumor mainly by bronchoscopy. Also, this strategy can be associated with systemic immunotherapy and SBRT	Immunomodulant can promote a change of tumoral microenvironment, which acquires a favorable immunological setting, becoming “hot”. At the same time some immunomodulants may directly destroy the tumor. The available trials define a good efficacy and safety profile.	There are only phase I and II trials. It requires the bronchoscopy that could be associated with possible complications and adverse events.
Intrapleural therapy	This strategy is HITHOC.	It may be a valid alternative to pleural effusion invasive strategies (evacuation, pleurodesis) especially in frail patients. HITHOC may be useful also in the oligometastatic setting of pleura.	We have scarce data about HITHOC in NSCLC and in this setting, it cannot be recommended [57].
Inhalation therapy	Antiblastic therapy can be administered via inhalation.	Dry powder form may increase drug exposure at the targeted tumor site with a smaller dose. As such, safety profile may be enchanced.	The administration can be difficult for patients. Severe chronic lung patology (e.g., COPD, asthma etc) are main controindications. Unfortunately, they are quite common in NSCLC patients because they share smoking as a risk factor.
Photodynamic therapy	PDT consists of a photosensitizer drugs, excited by appropriate wavelength laser irradiation.	It can be an extreme specific tumor site therapy without important complications. It may be used in palliative setting especially in multimodal treatment. Moreover, it may be used alone in very strict cases of early central NSCLC (unresectable).	PDT is not recommended for deeper lesions or too dense lesions (necrotic sites), because in these cases PDT would not be effective
Thermal ablation	Extreme temperatures (heat and cold) can induce tissue damage and provoke a modification of tumoral microenvironment.	This strategy can be used in palliative setting, especially in oligometastatic disease. In addition, it finds space in selected curative setting, if patient shows contraindication to surgery or stereotactic radiotherapy. Ablative therapies and immunotherapy may improve their efficacy mutually.	These ablative therapies show only one absolute contraindication (untreatable coagulopathy) and technical ones (tumor is too close to vital structures). The typology of thermal ablation should be selected accurately, also based on the physical conditions of cancer.
Brachytherapy	The local radiotherapy can be administrated with permanent implantation of I125 vycril mesh	Brachytherapy can be useful in palliative setting, especially if present important symptoms (dyspnoea, haemoptysis etc.). After the implant, quality of life and symptoms may improve quickly. Instead, the indications in radical setting are strictly selected and limited.	Brachytherapy depends on bronchoscopy; therefore, it is important to think about complications and contraindications. Moreover, it may have “rivals”, like ablative strategies.
Tumor treating fields	Alternating electric fileds with low-intensity and intermediate frequency can inhibit the tumoral cell growth	This strategy has shown interesting results about efficacy and tolerability, with a good safety profile.	The device is quite expensive and “lumbering” for the patient
Nanoparticles	Nanoparticles have a potential anti-inflammatory antioxidant and immunomodulatory effect. In addition, nanoparticles are perfect carriers, which can bring not only traditional antiblastic agents, immunotherapy and radiotherapy, but also non-conventional elements.	They have a cross role with other intratumoral and systemic strategy. So they may be a future cornerstone.	Despite their important impact, we have only I and II phase trials.

## Data Availability

Data are contained within the article.
